# A Systematic Review of Virtual Reality’s Impact on Anxiety During Palliative Care

**DOI:** 10.3390/healthcare12242517

**Published:** 2024-12-12

**Authors:** Alexandra-Maria Gaina, Cristinel Stefanescu, Andreea-Silvana Szalontay, Marcel-Alexandru Gaina, Vladimir Poroch, Daniela Viorica Mosoiu, Bogdan-Victor Stefanescu, Magdalena Axinte, Cristina-Maria Tofan, Liviu Adrian Magurianu

**Affiliations:** 1 Department of Medicine III, Faculty of Medicine“Grigore T. Popa”, University of Medicine and Pharmacy of Iasi, 16 Universitatii Street, 700115 Iasi, Romania; alexandra_gaina@email.umfiasi.ro (A.-M.G.); cri.stefanescu@umfiasi.ro (C.S.); andreea.szalontay@umfiasi.ro (A.-S.S.); vladimir.poroch@umfiasi.ro (V.P.); bogdan-victor.c.stefanescu@students.umfiasi.ro (B.-V.S.); 2 Romanian Academy, Carol I Boulevard 8, 700506 Iași, Romania; magda.axinte@gmail.com (M.A.); cmtofan@acadiasi.ro (C.-M.T.); liviu.magurianu@acadiasi.ro (L.A.M.); 3 Institute of Psychiatry “Socola”, 36 Bucium Street, 700282 Iasi, Romania; 4 Romanian National Palliative Care Association, “Grigore T. Popa” University of Medicine and Pharmacy, Main Building A1, 16 Universitatii Street, 700115 Iasi, Romania; daniela.mosoiu@hospice.ro; 5 Department of Palliative Care, Regional Institute of Oncology, 700483 Iasi, Romania; 6 Faculty of Medicine, Transylvania University of Brașov, Eroilor Boulevard 29, 500036 Brașov, Romania; 7 HOSPICE Casa Speranței, Sitei Street 17A, Brasov 500074, Romania

**Keywords:** virtual reality, palliative care, anxiety management, non-pharmacological treatments, quality of life, systematic review

## Abstract

Background: Virtual Reality (VR) is an emerging non-pharmacological treatment for anxiety in palliative care patients. Anxiety, a common symptom in this population, has a significant effect on living standards. The aim of this systematic review is to evaluate the effectiveness of VR interventions in reducing anxiety and improving quality of life in palliative care. Methods: The systematic review protocol was registered in PROSPERO (CRD42024517914). The comprehensive search was performed in nine databases, including PubMed, PsycINFO, and EMBASE, and included studies published up to 15 November 2024. RCTs, pilot studies, and feasibility trials involving adult palliative care patients in which VR interventions were used, were compared with standard care. Two reviewers independently extracted and assessed the quality of the data using the RoB 2 tool. Results: A total of 13 studies were considered eligible, with 333 participants aged 15 to 85 years old. In these experiments, anxiety decreased steadily, and in some cases mood and quality of life were improved significantly. The VR interventions ranged from guided nature walks to memory-training sessions. Sessions lasted between 5 and 30 min and ranged in duration from single sessions to daily use. Personalized and participatory VR-based content was particularly effective in alleviating anxiety. The heterogeneity of the study designs and VR protocols hampered meta-analysis, despite promising findings. Conclusions: VR has considerable potential as an adjunctive anxiety therapy for palliative care. The personalization and immersion that VR provides are psychologically unique and have the potential to lower anxiety and improve well-being. But standardizing intervention protocols and more studies are required to define the best VR strategies and evaluate outcomes over the long term. The article highlights the promise of VR as part of palliative care as a viable bio-psycho-socio-spiritual approach.

## 1. Introduction

Anxiety is a symptom often encountered in palliative care that negatively impacts the patient’s quality of life. Management options, usually pharmacological, are constrained by side effects and individual responses. More recent work indicates that the use of technological means as complementary treatments should be a more integrated part of treating anxiety in the palliative care setting. Interventions should focus on improving the quality of life psychologically, physically, and socially while integrating spiritual meaning.

### 1.1. Positive Psychological Well-Being (PPWB) and Complementary Therapies in Palliative Care

The early palliative care (EPC) models involve PPWB elements, including hope, gratitude, and acceptance of death, which have been found to lower anxiety and enhance clinical outcomes in advanced cancer patients. These aspects of PPWB are cultivable with interventions and, as such, have a longer-term outlook than late-stage palliative care, which tends to use symptomatic short-term medication solutions [1]. In a study conducted in Vietnam, multidisciplinary treatments of anxiety and depression among cancer patients in palliative care was reported to be able to prolong patients’ lives [2]. In hospice patients, Watson’s Human Care Model in nursing care reduces anxiety and improves life quality with compassion and centered care [3].

Functional level and quality of life are negatively impacted by comorbid anxiety and depression in palliative care patients. Furthermore, nausea, exhaustion, and lack of appetite are more prone to coexist when patients encounter these mental problems. These mental health-related risk factors are widely known; therefore, clinicians plan interventions to reduce anxiety and depression and provide cancer patients with greater dignity and quality of life during palliative care by incorporating alternative or complementary interventions within the therapeutic plan [4].

Guided imagery relaxation, sometimes synergized with music therapy and other complementary interventions, has been shown to reduce anxiety and pain in palliative care patients. The mechanism of action is represented by a focus shift from catastrophizing toward the positive aspects and instead on what is good, which translates into greater well-being. Such non-pharmacological treatments can be part of a multidisciplinary palliative care system that provides physical and psychological support [5]. The roots of palliative care patients’ anxiety derive from emotional distress, financial stress, and limitations in social functioning. Working on these associated patient-related outcomes is essential for achieving a better quality of life. Female patients, in particular, are more vulnerable to anxiety and depression, therefore suggesting the need for gender-targeted interventions [6].

The shift to more comprehensive forms of anxiety treatment is evidence of the inclusion of psychological well-being, integrated care, and integrative therapies in palliative care. This model considers the psycho–physical–social care dynamic to support palliative care patients to live with dignity. While pharmacological approaches still dominate anxiety treatments, complementary approaches such as psychosocial interventions can improve outcomes for patients. This integrated approach is becoming mainstream based on palliative care aims: to lessen pain, either physical or psychological, and enhance well-being.

Integrative palliative care melds the practices of palliative care and integrative medicine, where whole-person care and well-being are central elements. It involves a wide variety of evidence-based therapies that are not pharmacological and are complementary to standard care, such as acupuncture and dietary supplements [7,8].

The final aim is to minimize physical, psychological, and spiritual distress by using all the available effective methods to enhance patients’ and their families’ quality of life [7].

Combining conventional and state-of-the-art medicine in healthcare systems is essential for effective holistic care. Collaboration in research studies, interdisciplinary training, and policy reforms would be necessary to support this integration in the face of problems such as standardization, regulation, and cultural adaptation [9].

### 1.2. Integrative Oncology: Yoga for Palliative Care Anxiety Management

Multimodal treatments—wherein multiple therapeutic strategies are used together—have been successful in the treatment of anxiety and depression. Such programs boost the quality of life and decrease patients’ reported symptom intensity. The programs are usually supported by a continuation-phase preventative measure and show significant longer-term benefits for patients [10].

Ancient Indian medicine, Ayurveda, provides a mind–body model for mental healthcare. It includes therapeutic methods like dietary changes, lifestyle changes, herbal medicine, and mind–body techniques. Incorporating Ayurveda in palliative care can complement traditional treatments and offer personalized, effective care for mental health problems such as anxiety and depression [10].

Integrative oncology offers science-based suggestions for anxiety and depression in patients with cancer. MBTI (mindfulness-based interventions), yoga, and relaxation therapy are also recommended before and after cancer therapy. Such interventions are part of a broader trend of incorporating alternative treatments into standard cancer treatment for psychological and physical symptoms [11].

Integrating holistic and integrative medicine in palliative care has many benefits that are counterbalanced by implementation challenges. For such add-on therapy to be integrated within the therapeutic protocols, patient–healthcare provider communication, health inequities, and costs must first be addressed in advance. Moreover, the evidence base for some integrative treatments is limited, so more research will be required to establish their efficacy and safety. Even with these setbacks, the transition to inclusive care results from a greater awareness of the need to consider palliative care’s psychological, physical, and social dimensions.

Yoga has become a useful adjunctive intervention for anxiety management in palliative care. The physical postures, breathing, and meditation of yoga provide a total approach to anxiety disorders that negatively impact palliative care patients because of the anxiety caused by the end of life and death.

Stress management: According to a meta-analysis of randomized controlled trials, yoga did have a statistically significant positive effect on anxiety symptoms (effect size 0.80), therefore representing a viable dual mind–body intervention for psychological symptoms [12].

Spirituality: Yoga does not target anxiety alone, but facilitates well-being by promoting physical fitness, mental resilience, and spiritual stability. It provides strategies for grief, caregiver burnout, and thanatic phobic aspects, common in palliative care [13].

Physical Impairment: Some types of yoga (Dru yoga) have been adapted to be inclusive to patients who have physical impairments and have proved suitable for inclusion within the therapeutic programs of patients in palliative care [14].

Resilience: Yoga regulates the body’s response to stress, one of the most important aspects of anxiety management. Practices like pranayama (breathing exercises) and dhyana (meditation) work well for stress reduction and emotional regulation [15].

In a brief yoga lifestyle intervention, anxiety levels were significantly reduced and subjective well-being was improved among chronic disease patients, suggesting that similar effects could be observed in palliative care patients [16].

Yoga can be combined with standard treatment as an alternative non-drug solution that can be part of a multidisciplinary palliative strategy. This aspect is essential for the care of both physical and mental symptoms in palliative care [5].

The medical application of yoga for anxiety is justified by the fact that it is cost-effective and accessible. The implementation of yoga into the palliative care protocols must be designed to be safe and practical for different ages and physical abilities of patients. Specialized yoga sessions designed around such needs can improve patient engagement and patient-reported outcomes [13].

Altogether, yoga can be a potential adjunct to the treatment of anxiety in palliative care in regard to its adaptability to patients’ needs and preferences, facilitating incorporation into existing care plans.

### 1.3. The Potential of Harnessing VR in Palliative Care

It has become possible to combine VR and gamification in palliative care to create a novel, non-pharmacological treatment to help relieve distress and improve motivation to enhance the quality of life for terminally ill patients. VR allows users to vividly experience computer-generated worlds, and their presence in natural VR environments, facilitated by sensory and emotional relaxation, can temporarily relieve physical and mental tension [17,18]. VR has become a more common form of treatment for pain and anxiety and of cognitive training in healthcare settings, particularly in the surgical and oncological environments. The simulated environment of VR provides distraction that can mitigate perceived pain during surgery and improve patient comfort [19]. The technology has proven to be effective in a range of settings in medicine, from pediatrics to oncology to surgery, though the efficiency varies by use and population.

VR has been implemented as a distraction tool for children during needle procedures to reduce pain and anxiety. A systematic review and meta-analysis reported strong reductions in pain and anxiety with VR, but high heterogeneity between studies [20]. Another meta-analysis found that VR significantly lowers pain scores in children during clinical visits [21]. Still, other studies found no improvement in anxiety relief or pain reduction when clinicians were using commercially available VR software versus routine practice in pediatric emergency rooms. This indicates the demand for more personalized VR solutions for children’s psychological needs during healthcare [22].

VR has been shown to help in reducing anxiety during surgical interventions. For example, when VR was employed in a pilot trial on patients undergoing head and neck surgery under local anesthesia, perioperative anxiety was significantly reduced, and most patients had a high level of adherence [23]. While VR was reported to be a promising treatment for the management of pain and anxiety in some cardiovascular surgery procedures and interventional cardiology studies, other trials reported no benefit [24,25].

VR has also been applied to reduce pain and anxiety during dental procedures like implant surgery, in which it was used to decrease pain and anxiety [26,27].

In oncological settings, VR has been used to control anxiety and pain for breast cancer patients; it was extremely effective in alleviating pain, but lacked statistical significance for anxiety alleviation [28]. In pediatric oncology, VR has been deployed to distract patients from pain and anxiety during traumatic procedures. It has been demonstrated to be a highly effective non-pharmacological pain reliever that may be combined with typical painkillers such as opioids [29].

In addition to pain and anxiety, VR has also been demonstrated to support patient education, engagement, and treatment compliance, leading to improved clinical outcomes [30].

VR treatments have been shown to enhance emotional well-being and decrease anxiety in palliative care patients. Individualized VR content such as virtual house visits can contribute to emotional touch and well-being, without increasing the psychosocial burden, while limiting cybersickness [31]. Moreover, VR therapy was shown to reduce physical and psychological suffering, which allowed patients to introspect, understand themselves, and find tranquility [32]. VR has been successfully used to ease symptom distress in palliative care patients. Research has also shown increased occupational performance and satisfaction and decreased distress caused by symptoms such as pain and fatigue [33]. Moreover, VR-delivered music therapy has demonstrated moderate clinical relief of physical and mental suffering [34].

Cooperative VR games have been proposed to lower anxiety and pain in pediatric patients while undergoing surgery. Such a strategy results in positive shared experiences and reassurance between children and caregivers; thus, VR is well positioned to potentially influence patient–carer relations positively [35].

Virtual reality (VR) is also being used in more general cancer treatment settings, and it has been shown to reduce anxiety and boost patient satisfaction during intensive care. For instance, Mitello et al. (2024) [36] and Li et al. (2024) [37] report that VR interventions alleviated anxiety in chemotherapy patients by reducing estimates of treatment duration, discomfort, and psychological distress. Although these experiments are not focused solely on palliative care patients, they do show VR’s promise as an infusion of distraction and emotional support across all phases of cancer care. This prescient evidence behind VR in earlier cancer treatments suggests it may be even more enticing to patients in palliative care, where stress management and quality of life are central targets. Also, the VR-for-mindfulness program SENTIR developed by Carneiro et al. (2023) [38], in which VR is integrated with mindfulness, reinforces VR’s efficacy for emotional resilience by offering quiet environments that can promote relaxation and stress reduction.

### 1.4. Challenges of VR Adoption in Palliative Care

Current research suggests that VR can be easily implemented into mainstream palliative care and could provide an efficient complement to conventional therapies [39].

Still, VR devices can also be perceived as a burden by the patient, impairing the presence, immersivity, and overall experience. The reasons for this perception are mostly the HMDs’ (Head-Mounted Displays) weight and design, leading to strain and therefore resulting in diminished patient participation. There is also the need for more immersive VR to create better user experiences. Addressing these issues causing patient discomfort during exposure results in a strong need for lighter HMDs and more interactive content to make them more comfortable and engaging [31,32,40].

The weight of VR headsets is an important aspect that affects the patient experience. For example, heavier headsets reduced comfortable wear time by an average of 11 min per 33 g [41]. Similarly, the correct weight distribution and balance also has an influence on fatigue and pain, and incorrect balance causes more pressure on the neck [42]. Different constructions and wearing conditions of VR devices can affect center of gravity and weight distribution, resulting in the subjective sensation of discomfort [43]. Integrated head-wear devices are generally more comfortable than HMD models with soft belts [44].

Privacy issues and whether VR can cause negative feelings like homesickness have been noted. However, personalized VR content has been demonstrated to be both possible and efficient, and privacy issues can be alleviated by involving the family in content creation [31].

VR has promise for palliative care, but its psychological dangers must also be taken into account. There are patients who can suffer from discomfort or negative psychological outcomes, including homesickness or depression, after VR experiences. Therefore, psychological evaluations prior to VR therapy are essential, and subsequent interventions should also be provided to minimize these risks [45].

The vergence–accommodation conflict also causes eye discomfort, as the eyes must adapt to different focal distances, therefore becoming strained. Solutions such as focus-adjustable lenses have been shown to overcome this, leading to an overall increase in comfort [46].

The weight-to-functionality trade-off is an important consideration, because functionality usually means more weight, and more weight leads to less comfort [41]. Furthermore, VR systems still require lower costs and complexities in user interfaces to promote wider adoption and implementation in healthcare [40,47].

### 1.5. Objectives

This systematic review aims to analyze the impact of VR for anxiety in palliative care settings while exploring the types of interventions employed, assessing their effectiveness, and highlighting the unique elements that contribute to VR’s impact, such as the level of immersion, content customization, and frequency of use. By synthesizing findings across multiple studies, this review could provide a comprehensive perspective on VR’s potential as a non-pharmacological approach for anxiety relief in palliative care, offering insights into its benefits and limitations. The ultimate objective is to inform clinical practices and encourage further research into VR as a viable, evidence-based option in the palliative care setting while addressing the following questions:

(A) Do VR treatments significantly impact anxiety in palliative care patients?
(B) What are the types of VR interventions that have been used in palliative care, and have they been effective at reducing anxiety?
(C) What specific design features of VR therapy (personalization, interactivity, session frequency) seem to sustain or enhance therapeutic performance in the palliative care environment?

## 2. Materials and Methods

### 2.1. Protocol Registration

This systematic review protocol was registered on PROSPERO on 8 March 2024, under ID CRD42024517914. This review adheres to the PRISMA 2020 guidelines to ensure methodological transparency and reproducibility [48].

### 2.2. Eligibility Criteria

The study inclusion and exclusion criteria were defined using the Population, Intervention, Comparator, Outcome, and Study Design (PICOS) framework, as revealed in Table 1, according to recommendations of the Cochrane Handbook for Systematic Reviews of Interventions, Version 6.2 [49].

### 2.3. Search Strategy

A systematic search was conducted across nine databases, PubMed/MEDLINE, PsycINFO, EMBASE, CINAHL, Cochrane Library, PsycARTICLES, Scopus, Web of Science, and ProQuest, up to 7 November 2024. Boolean operators were adjusted based on database requirements, as revealed in Table 2.

### 2.4. Study Selection Process

Two researchers (A.M.G. and C.M.T.), while working independently, conducted a thorough examination of full-text documents and identified studies that met the eligibility criteria. Disagreements were amicably resolved through deliberation or consultation involving a third author (L.A.M.), ultimately leading to consensus.

### 2.5. Data Extraction, Synthesis, Outcomes, and Statistical Analysis Feasibility

A standardized data extraction form was developed and used to ensure consistent data collection across studies. The following information was systematically extracted from each eligible study, as detailed in Table 3: study design, participant demographics, VR intervention, and outcomes.

Data retrieved from eligible studies were extracted narratively due to the included studies’ design heterogenicity reflected different designs, interventions, populations, and outcome measurement parameters, which precluded meta-analysis.

### 2.6. Risk of Bias Assessment

Each study was subjected to a rigorous Risk of bias 2 (RoB2) analysis to evaluate its methodological quality and reliability, while considering the following five domains: randomization procedure, divergence from interventions, missing data, outcome measurement consistency, and selective reporting [50].

Every area was classified as having a “low risk”, “some concern”, or “high risk” of bias. More than one domain deemed “high risk” represented the cutoff for a study to be flagged for exclusion, as the identified biases seriously undermined the reliability of the outcomes.

In an attempt to adjust for reporting variance, papers that were assigned “some concerns” were peer-reviewed in terms of their total contribution to shaping broader findings and to avoid disproportionate and unnecessary exclusions that might bias conclusions.

## 3. Results

### 3.1. Study Selection and Characteristics

**Figure 1:**
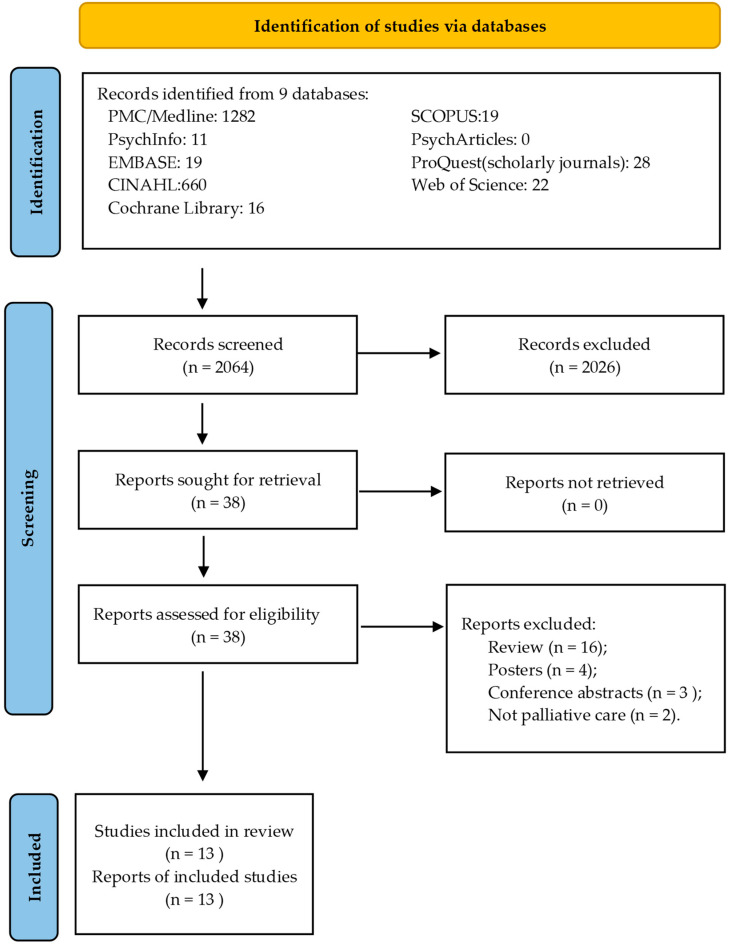
Prisma diagram. Figure 1 was adapted from [48]. For more information, visit: http://www.prisma-statement.org/ accessed on 28 April 2024 [28].

The systematic review includes 13 studies (RCTs, pilot studies, and feasibility trials) as presented in the PRISMA flowchart (Figure 1), totaling 333 participants. These patients, aged between 15 and 85, were mainly in palliative or hospice care, and the majority had advanced cancer.

Along with anxiety as an outcome, other psychological and physiological measures such as pain, quality of life, and existential crisis potential were also assessed [48].

These studies were conducted in diverse geographical regions, ranging from North America to Europe and Asia, each of which provided a different cultural context for VR application in palliative care. It varied across VR interventions, from naturalist simulations and mental imagery to calming scenes and synchronized VR experiences for patient and caregiver. Sessions ranged in length from 5 to 30 min, and the number of exposures varied from single sessions to daily interventions over weeks. This range shows VR’s potential for accommodating varying therapeutic needs and preferences in palliative care.

The gathered data are heterogeneous due to variations in their design, patient population, VR content, and outcomes; therefore, generalization of their results is challenging. Due to the gathered data heterogenicity, meta-analytical statistical processing was not deemed feasible.

The variation in study design, patient populations, and VR protocols reflects VR’s versatility as an alternative tool in palliative care. Even with these differences, these findings converge to indicate VR’s potential to emerge as a non-pharmacological approach to anxiety, psychological functioning, and overall quality of life in palliative care patients, as depicted in Table 4.

### 3.2. Anxiety Reduction and Emotional Relief

One common finding across all studies we included was VR’s capacity to reduce palliative care patients’ anxiety. For instance, Burridge et al. (2023) [55] showed the most significant reduction in anxiety, as the mean anxiety score declined by roughly 40% post-intervention, from a baseline of 4.43 (SD = 2.56) to 2.65 (SD = 2.24). Similarly, Schrempf et al. (2022) reported reduced stress levels from self-reports as well as lower heart and respiratory rates after VR sessions [53]. These physiological indicators also supported patients’ post-intervention descriptions of feeling more content and relaxed.

Nwosu et al. (2021) [52] used VR treatments to expose patients to scenes of natural environments, including beaches and forests, which 93 percent of the study participants described as improving their mood. What is more, patients were generally accepting and excited about future VR sessions, which indicates that VR can be a practical and attractive treatment for anxiety in this patient group [52].

Moscato et al. (2021) [57] reported significant decreases in anxiety and pain in palliative care patients following personalized VR interventions; the VR sessions set in familiar surroundings achieved significant patient-related outcome improvements, suggesting VR’s utility as an individualization strategy in hospice settings. Similarly, Deming et al. (2024) [61] demonstrated a 30% reduction in anxiety scores for hospice patients after watching peaceful nature VR videos, which led to improved mood and psychological relief post-intervention.

### 3.3. Content and Personalization Impact on Results with VR

According to the research, content and personalization were the most critical factors in making VR experiences effective. According to Lloyd and Haraldsdottir (2021), virtual reality regressions to homes from childhood or to dream destinations were successful in reducing anxiety and improving moods in the patients [51]. Such individualized VR content made it easier for patients to feel immersed and therefore satisfied, with patients often referring to the experiences as imbued with an affective framework, such as being “therapeutic” or “comforting” [51].

Other experiments using less personalized VR experiences—guided meditation, for example, or general nature scenes—were similarly practical. Still, they had slightly lower emotional impact and satisfaction than the customized version. Kupczik et al. (2022) [56] found that 3D environments that allowed patients to touch, feel, and manipulate aspects of the VR environment, like picking up objects or experiencing virtual nature, engaged and satisfied patients more than passive 360-degree video.

Greinacher et al. (2024) [59] collected qualitative responses that showed that dying cancer patients found VR distracting, calming, and emotionally uplifting, and believed it was a tool of psychological stress relief. McAnirlin et al. (2024) [60] concluded that the Tandem VR™ protocol, tailored for both hospice patients and caregivers, alleviated psychological suffering while improving emotional care for both. This dual-user approach also demonstrated how VR might be able to reduce both caregiver and patient anxiety.

### 3.4. VR Perspectives in Palliative Care

Acceptance and feasibility were also generally high across the studies, indicating that VR treatments are tolerated and achievable for palliative care patients. Guenther et al. (2022) [62] reported that 82.5 percent of patients wanted to participate in more VR sessions, and most did not report any side effects. If minor discomforts were present—the headset was too heavy, or the image was too blurry—these device-related aspects were corrected easily. VR interventions were, in most cases, brief and adaptable, so they were very easily integrated into the routines of palliative care [62]. With compassionate mind training in VR, O’Gara et al. (2022) [54] demonstrated VR’s psychological efficacy beyond relaxation by statistically enhancing mood and mental health.

VR was complementary to conventional palliative care protocols, being perceived as desirable by patients and providers alike as a treatment alternative that did not conflict with existing treatment or medication [62].

### 3.5. The Physiological and Psychological Signatures of Relaxation

A few studies assessed the physiological and psychological indicators of relaxation, supporting VR’s effect on anxiety reduction. Schrempf et al. (2022) [53] reported statistically significant decreases in heart and respiratory rates, encompassing reductions of 1.3 beats per minute and 0.6 breaths per minute, respectively. Such physiological shifts matched self-reported reductions in anxiety and improvement in relaxation and mood. These physiological markers serve as a scientific proxy for the effect of VR on relaxation, consistent with patients’ claims of lower psychological tension and better mood [53].

Burridge et al. (2023) [55] also found a relationship between VR and a decrease in anxiety on the 10-point Likert scale. They often described VR as an “evasion” from physical pain and mental suffering by patients. These physiological markers back up the subjective anxiety-lowering effect, suggesting VR could affect psychological as well as autonomic mechanisms [55].

Brungardt et al. (2024) [34] and Kelleher et al. (2022) [58] both reported VR’s impact on symptom burden and life quality. In the Brungardt trial, 25 min VR music sessions moderately improved symptom distress and quality of life in palliative care patients. Furthermore, Kelleher et al. (2022) [58] observed significant reductions in pain and anxiety following VR sessions in advanced colorectal cancer patients, indicating VR as an potential treatment for pain.

### 3.6. Limitations of VR Interventions

Although strong evidence supports the impact of VR in palliative care-encountered anxiety, issues about how VR could be implemented in palliative care arise. Research suggests that VR headsets might be too cumbersome for patients with limited mobility or severe physical disability.

Nwosu et al. (2021) [52] listed logistical issues, including infection control and maintenance of the VR devices, for hygiene and clinical usage.

Additionally, Guenther et al. (2022) [62] suggested better ergonomic and lighter VR systems that could accommodate the physical restrictions of the palliative care patients.

The other limitation across the studies was the degree of heterogeneity of VR intervention design in terms of content, time, and frequency. This heterogeneity hinders the meta-analyses needed for guideline recommendations. While this review is malleable in combining multiple findings, a standardized protocol of VR interventions may represent the silver bullet toward extending VR’s palliative care efficacy into clinical practice implementation.

For the majority of studies, the RoB was low regarding randomization and intervention protocol implementation, with attention to missing data and robust protocols for measuring outcomes. Yet some studies revealed subtle flaws in selective reporting, especially when it came to long-term psychological effects, as revealed in Table 5.

## 4. Discussion

These results promise that VR could alleviate mental suffering, offer emotional support, and foster patients’ resilience. However, the results also show significant variations in study design, patient populations, VR exposure digital content, and outcome measurement, stressing that research in this field must be standardized and methodologically robust. Furthermore, we will expose the highlights, discuss what current VR interventions offer and lack, and suggest a manner to surmount challenges impeding VR’s implementation in palliative care.

The results reported by Moscato et al. (2021) [57] and Gerlach et al. (2024) [31] emphasize the efficacy of VR as an individualized, non-pharmacological form of psychological support. These studies argue that by adjusting VR experiences to the needs of patients, VR can provide a distinct form of emotional safety, which can help patients live a better life without sensorially overburdening patients in palliative care. That personalization, such as offering environments tied to personal memories or calming nature settings, is particularly important in palliative care, where treatment must specifically focus on patient needs.

McAnirlin et al. (2024) [60] proposed a novel protocol targeting both patient and caregiver anxiety. This dual approach demonstrates VR’s wider role in palliative care by serving families and caregivers, thereby diminishing general psychological distress in hospice care. By sharing a mutual positive experience, VR can improve patient–carer relationships and strengthen the psychological resilience of both involved parties.

Kelleher et al. (2022) [58] and Deming et al. (2024) [61] demonstrate VR’s ability to provide significant reductions in pain and anxiety that might be as effective or better than conventional palliative care treatments such as counseling and meditation while also offering rapid results. When combined with conventional treatment, VR becomes an adaptive, person-centered intervention, raising the bar of analgesia and anxiolysis without exceeding pharmacological safe dosages that prone patients to side effects. Evidence from Mitello et al. (2024) [36] and Li et al. (2024) [37] demonstrate that VR treatments have efficacy for symptom relief and psychological comfort even in the absence of palliative care, thus making VR versatile for cancer care in general. In these experiments, we see reductions in anxiety and stress levels, and general patient satisfaction while undergoing chemotherapy treatment, which bolsters VR’s utility as a support tool. As alleviation of distress and existential anxiety are paramount, VR’s meditative, immersive nature can provide mental and emotional relief and assist in overcoming end-of-life stressors. Additionally, the mindfulness component in the SENTIR program (Carneiro et al., 2023) [38] suggests that VR and relaxation interventions could potentially provide additional psychological support for palliative patients by simultaneously reinforcing immersion and mindfulness.

### 4.1. VR Impact Beyond Anxiety

Results repeatedly revealed that VR interventions could alleviate anxiety in palliative care patients. Research such as Burridge et al. (2023) [55] and Schrempf et al. (2022) [53] reported measurable declines in subjective anxiety scores, and improved physiological relaxation measures including heart and respiratory rates, following VR sessions [53,55]. Such results are consistent with those in general medical research, where VR has been shown to offer psychological relief through immersive distraction by shifting sensory and cognitive focus. However, VR can create calming scenes or virtual trips to locations that are particularly helpful in providing patients with a short-term respite from suffering. Such immersion is necessary because it affords a kind of escapism that provides temporary relief and psychological sanctuary from physical and mental pain that terminally ill patients seek.

Even when findings are generally good, the lack of consistency between researchers regarding VR content and protocol means it is difficult to generalize conclusions in regard to the optimal layout of VR interventions. For example, while some studies emulated highly interactive VR experiences with flexible personalization, others were passive, non-interactive experiences such as 360-degree videos of natural environments. It is conceivable that personalized VR experiences, or those that specifically targeted patients’ interests or uncovered important personal memories, confer better emotional value because patients often felt comforted and at ease when vividly experiencing personalized content. More research should investigate where this optimum between customization and standardization lies to find which content features significantly impact anxiety alleviation.

### 4.2. Feasibility and Acceptability of VR Interventions in Palliative Care

What is most striking about VR across studies is the highly reported patient acceptability and clinical implementation feasibility. Research like Nwosu et al. (2021) [52] and O’Gara et al. (2022) [54] demonstrated that most patients were satisfied with VR and requested additional exposure sessions; they were delighted and willing to try VR as part of the treatment protocol. That embracing of VR exposure by the patients is crucial since it enables VR to be a real-world intervention used in daily treatment routines without overburdening patients or caregivers. The fact that VR is a non-pharmaceutical treatment is another advantage, as it presents a therapy that will not disrupt current gold standard approaches.

### 4.3. VR Interventions to Address “Total Pain”

These physiological responses to VR therapy, such as decreased heart and breathing rate, suggest that VR could impact patients beyond relieving anxiety [53,53]. As VR induces a focused, relaxed mood, it could be able to reduce other palliative care-associated symptoms such as pain, depression, and psychological distress. Research such as that by Guenther et al. (2022) [62] reported that VR immersion allowed patients to distract themselves for short periods from pain, a beneficial practice on all four biological, psychological, social, and spiritual dimensions when suffering from pain.

Attempting to treat the psychosocial demands of palliative care involves strategies that go beyond symptom avoidance and into existential suffering [63,64], as Kremeike et al. have suggested (2020) [65]; though not dedicated to VR, Kremeike’s research points to the necessity of offering patients the kind of information that will lessen the psychological burden of end-of-life issues. VR can help in this by fostering peaceful, meaningful virtual spaces that invite relaxation and reflection. Virtual reality can be both a therapeutic and psychological comfort for palliative patients, potentially helping to lower anxiety levels while creating moments of peace and psychological escape [66].

Having the patient virtually go back to places they care about or reminisce about pleasant memories acts like a psychological anchor for the suffering patient, and it can reduce the existential distress that end-of-life situations often lead to. This psychological advantage is borne out by the experience of patients in studies such as Lloyd and Haraldsdottir (2021) [51], in which VR allowed patients to reconnect with their history, and rely on expertise to create environments similar to something they already experienced. These effects suggest that VR might not be merely a distraction but an effective intervention promoting a sense of belonging and familiarity for palliative care patients.

### 4.4. Limitations

For all VR’s promise in palliative care, the current state of the art has limitations, principally in the study design variability and small sample sizes. The differences between VR interventions in content, session time, and interactions make it impossible to standardize and conduct a meta-analysis. Most experiments did not have control groups or large enough sample sizes to arrive at statistically significant results, but were included due to their pilot nature. These limitations suggest that future studies should adopt common protocols that are easy to replicate and compare, perhaps with larger randomized populations and the same psychometric outcome measures.

Another limitation is patient selection bias—studies typically include predisposed patients ready for a new technological treatment. Such a bias can lead to disproportionately positive outcomes and restrict generalizability to a wider palliative population. The subsequent studies need to include a more representative sample of palliative patients, including those who might be initially resistant to VR, to assess whether VR would be helpful in various populations. Lastly, VR was mainly well tolerated, but some patients did report discomfort or mild side effects like dizziness or trouble becoming used to the headset. These physical constraints will require newer, lightweight VR headsets and improved interfaces. Research must look at the optimal frequency and duration of VR experiences to balance efficacy with patient comfort and safety.

Lighter headsets such as the Samsung Gear VR or standalone Oculus Go worked best in portable and deployment-friendly scenarios, whereas tethered devices (i.e., HTC Vive) worked better for static, immersive, computationally demanding scenes.

The VR headsets used in the included studies varied in technical sophistication and design to suit the specific circumstances of the palliative care patient. Oculus Rifts and HTC Vive high-end HMDs were employed in the experiments of Schrempf et al. (2022) [53], and Burridge et al. (2023) [55] because of their superior motion tracking (6 DoF) and high-resolution immersive experiences due to being powered by dedicated graphics processing units of personal computers These headsets complemented both active interventions (guided meditations and breathing exercises) as well as passive ones (immersive nature simulations), so they were useful tools for addressing anxiety and stress in palliative care.

Other research such as that of Moscato et al., 2021 [57] or Deming et al., 2024 [61] prioritized lightweight, more accessible VR headsets such as Samsung Gear VR and Oculus Go, with 3 DoF tracking. An important aspect is that the Samsung Gear VR, currently discontinued, uses a smartphone, therefore offering a low-end immersion while promoting accessibility at lower costs. These were also chosen for ergonomics and user-friendliness, especially in the case of patients with physical limitations or in the late stages of disease. They were lighter and simpler, reducing patient fatigue and facilitating easy connectivity with intuitive platforms like TRIPP and Relax VR, which provide guided meditations and personalized virtual experiences based on patient choice.

Higher-end headsets such as the successors of the Oculus Rift (Oculus Rift S) and HTC Vive (HTC Vive Pro 2) cost around USD 399–USD 999, depending on peripheral accessories such as controllers or sensors, making them less accessible for mass adoption in resource-constrained environments.

The middle-priced headset Oculus Go was discontinued in 2020, while the current price of the Meta Quest 3, which offers mixed-reality interaction possibility ranges from USD 499, with the limited mixed-reality potential Meta Quest 3s starting from USD 299.

Content licensing, customization, and peripheral devices (headphones, sanitation kits) further add to the costs—setup fees are estimated between USD 500 and USD 2000 per institutional unit, while some software may need monthly or yearly subscriptions.

Hygiene is a logistical issue, particularly for immunocompromised patients. The disposable face masks and antimicrobial disinfectant products are incremental costs but are critical for hygiene.

These studies reported varying levels of patient VR tolerance, especially in elderly adults who have little or no exposure to technology. They sometimes needed to be trained in their use by patients and carers, which also takes time and resources.

The diversity of VR headsets shows an evolving technology, but a sense of how the technical capabilities intersect with the usability is important for palliative care. Advanced HMDs offer better immersion but are expensive and difficult to transport, which may limit their use in environments with limited resources. The lighter, more accessible headsets represent a realistic alternative that would enable more patients to participate and deliver the same therapy. More research must be performed to assess cost-effectiveness, usability, and long-term effects to help guide the adoption of VR into routine palliative care.

### 4.5. Future Directions and Implications for Practice

VR must be introduced into palliative care with appropriate sensitivity to ethical concerns related to data and patient privacy so that the application is safe and efficient [67]. Further research and technological development will likely increase VR’s utility as a tool in mental healthcare, opening up new possibilities for anxiety reduction and symptom relief [68]. With advancing technology like VR, palliative care can be revolutionized by providing complementary non-pharmacological interventions to enhance patient outcomes and quality of life. VR is a novel non-pharmacological strategy for alleviating anxiety and improving mental health in palliative care. This systematic review suggests that VR may help with psychological stress and induce a feeling of serenity, that patients are very likely to adopt it, and that it is clinically viable. However, the cross-study variability underscores that more research is needed to develop uniform protocols, make the devices more straightforward to use, and better understand VR’s broader therapeutic potential. As the technology evolves, VR can also become part of palliative care and provide patients with relief and consolation in their last moments.

Reported results from research such as Moscato et al. (2021) [57], McAnirlin et al. (2024) [59], and Greinacher et al. (2024) [60] points to VR’s potential as a flexible, individualized emotional support system, which confers unique advantages and fits in with palliative care’s overall aims [57,59,60].

With VR’s established promise, future studies must focus on adapting VR protocols to wider acceptance and individualized application in palliative care. Studies like McAnirlin et al. (2024) [60] point to VR’s potential for group interactions that offer caregiver support, which pave the way for multi-user VR for hospice care. Handling the practical problems that Brungardt et al. (2024) [34] notes will accelerate VR’s integration into palliative care.

The results underscore VR as an accessible, low-risk intervention with wide-ranging potential to help with symptoms and anxiety in palliative care. By integrating VR with other therapeutical modalities, health professionals can offer integrated psychological care that increases the quality of life and well-being of patients in palliative care [34,61].

Further studies are needed to investigate the full extent of VR’s biopsychosocial effects in palliative care [68,69].

The growing body of literature suggests that VR has the potential to mitigate anxiety by offering relaxation, distraction, and relief from the confinements of illness. For instance, studies like those by Lloyd and Haraldsdottir (2021) [51] and Schrempf et al. (2022) [53] document anxiety reduction and mood improvement among patients who engage in immersive virtual experiences. These studies indicate that VR may foster an environment where patients can virtually visit serene locations, experience comforting sensations, or reconnect with positive memories. Some VR programs in palliative care have taken this concept further by tailoring experiences that replicate places of significance for patients, enabling them to reconnect with cherished memories or fulfill “bucket list” aspirations virtually, which may otherwise be inaccessible due to physical limitations.

Research in the past decade has expanded from small, exploratory studies to RCTs and service evaluations, each adding dimensions to our understanding of VR’s impact in this setting. However, the existing research remains varied in its approach, with significant heterogeneity across studies regarding VR’s content, duration of exposure, outcome measures, and the specific patient populations examined. Such variability presents challenges for synthesizing data and drawing universal conclusions, thus highlighting the need to assess the current state-of-the-art evidence, identify efficacy patterns, and clarify VR’s role in anxiety management within palliative care [51,53].

This review consolidates the promise of VR for use in palliative care while revealing essential areas where more research is required to develop the most appropriate implementation framework. To make VR an institutionalized part of palliative care, future research should address the following:

- Standardization of VR RCTs protocols design: Establishing evidence-based standardized parameters regarding session length, frequency, and content development could enhance the reproducibility and comparability of VR interventions across studies and clinical settings.
- Assessment of the long-term effects of VR exposure: Investigating the persistence of VR’s impact on anxiety, mood, and overall well-being will provide insights into its effectiveness as a long-term symptom management tool in palliative care.
- Multi-symptom applications: Beyond anxiety and physiological relaxation, VR’s potential to address pain, depression, and existential distress warrants exploration to expand its utility in palliative care.
- Improving accessibility and usability: Developing VR systems tailored to the needs of older adults and physically impaired patients will increase comfort and safety and broaden adoption in palliative care environments.

## 5. Conclusions

VR has proven potential to represent a promising non-pharmacological approach to anxiety reduction in palliative care. This systematic review highlights the potential of VR to improve patient quality of life and promote psychological resilience. The high level of user acceptance indicates feasibility for implementation within palliative care treatment protocols. Still, prior to uptake within healthcare as a complementary intervention, future VR research must address the following:

- Standardizing intervention protocols and developing uniform VR intervention guidelines to enhance comparability and reproducibility across studies;
- Long-term impact studies investigating the sustained effects of VR on anxiety and other related symptoms to understand its long-term benefits in palliative care;
- Enhancing device accessibility by focusing on the ergonomic design of VR devices to accommodate the unique needs of palliative care patients, ensuring that interventions are accessible and practical for all patients.

By addressing these points, VR can evolve into a widely implemented complementary intervention in palliative care, potentially transforming it into a fundamental component of holistic patient management. These results point to VR as a multi-symptom treatment that could lower medication doses or represent an adjunctive therapy to address “total pain”, as coined by Cicely Saunders, enriching the quality of life and care for those with life-limiting conditions [70].

## Figures and Tables

**Table 1:** PICOS framework for study inclusion and exclusion criteria.

Criteria	Inclusion	Exclusion
Population	Patients receiving palliative/hospice care.	Studies not focused on palliative care settings.
Intervention	Receiving immersive VR HMD interventions.	Non-immersive VR interventions.
Comparator	Standard care, waitlist, placebo, or other non-VR interventions; single-arm pilot trials.	Studies without a comparator group or with insufficient control conditions (e.g., case reports).
Outcome	Anxiety, stress, or psychological distress assessed via validated psychometric tools.	Studies without outcomes related to anxiety, stress, or psychological distress.
Study design	Randomized controlled trials (RCTs), pilot studies, or feasibility trials published in peer-reviewed journals.	Observational studies, conference abstracts, posters, reviews, commentaries, editorials, or letters.
Language	Studies published in English due to resource constraints for accurate translation.	Non-English publications.

**Table 2:** Search strategy.

Database	Search Phrase Terms
PubMed/ MEDLINE	(“virtual reality”[MeSH] OR “virtual reality” OR “VR” OR “immersive experience” OR “computer-generated environment” OR “simulated environment”) AND (“anxiety”[MeSH] OR “anxiety” OR “psychological distress” OR “stress” OR “fear”) AND (“palliative care”[MeSH] OR “palliative care” OR “hospice care”[MeSH] OR “hospice” OR “end-of-life care”)
PsycINFO	(“virtual reality” OR “VR” OR “immersive technology” OR “simulated environment” OR “virtual therapy”) AND (“anxiety” OR “fear” OR “psychological distress” OR “mental stress”) AND (“palliative care” OR “hospice” OR “end-of-life care” OR “terminal care”) AND (“outcome” OR “impact” OR “effect” OR “efficacy” OR “effectiveness”)
EMBASE	(‘virtual reality’/exp OR ‘VR’ OR ‘immersive technology’ OR ‘simulated environment’ OR ‘computer simulation’) AND (‘anxiety’/exp OR ‘psychological distress’ OR ‘fear’ OR ‘stress’) AND (‘palliative care’/exp OR ‘hospice care’ OR ‘end-of-life care’ OR ‘terminal care’) AND (‘treatment outcome’/exp OR ‘efficacy’ OR ‘effectiveness’ OR ‘impact’)
CINAHL	(MH “Virtual Reality” OR “VR” OR “immersive technology” OR “virtual environment”) AND (MH “Anxiety” OR “psychological distress” OR “fear” OR “stress”) AND (MH “Palliative Care” OR “hospice” OR “end-of-life care”) AND (MH “Treatment Outcomes” OR “effect” OR “efficacy” OR “impact”)
Cochrane Library	(virtual reality OR immersive technology OR virtual environment) AND (anxiety OR psychological stress OR fear) AND (palliative care OR hospice OR end-of-life) AND (outcome OR impact OR effect OR efficacy)
PsycARTICLES	(“virtual reality” OR “VR” OR “immersive technology”) AND (“anxiety” OR “psychological distress” OR “fear” OR “stress”) AND (“palliative care” OR “hospice” OR “terminal care” OR “end-of-life care”) AND (“effectiveness” OR “efficacy” OR “outcome” OR “impact”)
ProQuest Psychology Database	(“virtual reality” OR “VR” OR “immersive technology”) AND (“anxiety” OR “stress” OR “fear” OR “psychological distress”) AND (“palliative care” OR “hospice” OR “end-of-life care”) AND (“outcome” OR “impact” OR “efficacy” OR “effectiveness”) AND (“clinical trial” OR “randomized controlled trial” OR “case report”) NOT (“literature review” OR “systematic review” OR “meta-analysis”)
Web of Science	(TS = (“virtual reality” OR “VR” OR “immersive technology”)) AND (TS = (“anxiety” OR “psychological distress” OR “fear” OR “stress”)) AND (TS = (“palliative care” OR “hospice” OR “end-of-life care”)) AND (TS = (“outcome” OR “impact” OR “efficacy”))
Scopus	(TITLE-ABS-KEY(“virtual reality” OR “VR” OR “immersive technology”)) AND (TITLE-ABS-KEY(“anxiety” OR “stress” OR “fear” OR “psychological distress”)) AND (TITLE-ABS-KEY(“palliative care” OR “hospice” OR “end-of-life care”)) AND (TITLE-ABS-KEY(“outcome” OR “impact” OR “efficacy” OR “effectiveness”))

**Table 3:** Standardized data extraction form.

Category	Detail Sheet
Study description:	Author, publication year, study design (e.g., RCT, pilot study, feasibility trial)
Participant demographics:	Number of participants, age group, population (i.e., advanced cancer, hospice care)
VR intervention:	VR hardware, software, type of VR, number of sessions, duration of interventions, customization
Anxiety-linked outcomes:	Psychometric outcome measures (e.g., Hospital Anxiety and Depression Scale [HADS], Generalized Anxiety Disorder-7 [GAD-7]) and other anxiety-specific outcome measures
Secondary outcomes:	Other secondary outcomes, like pain relief, improved quality of life, and physiological parameters (e.g., heart and/or respiratory rate)

**Table 4:** Overview of virtual reality interventions for anxiety and pain management in palliative care studies.

Authors	Study Design; Participants (n *)	VR Intervention: Hardware, Software, Duration	Psychometric and Physical Evaluation of Anxiety
Gerlach et al. (2024) [31]	Feasibility study; inpatients in palliative cancer care (*n* = 12)	VR with personalized settings such as patient-selected home environments; 15–20 min per session.	Anxiety and well-being assessed pre- and post-intervention; reported significant increase in comfort with reduction in emotional burden and anxiety levels.
Brungardt et al. (2024) [34]	Pilot study; hospitalized palliative care patients (*n* = 20)	VR HMD combined with music therapy; 25 min sessions using serene auditory–visual experiences.	Symptom distress measured with quality-of-life scores improved moderately; psychological stress and anxiety reduced based on self-report scales.
Lloyd and Haraldsdottir (2021) [51]	Qualitative study; VRG (*n* = 19)	30 min VR session using “room-scale” VR with participant-chosen locations (e.g., childhood home, holiday spots). Guided by an experienced facilitator to create personalized experiences.	Positive impact observed: Participants reported fulfilling experiences and meaningful memories. Mild negative responses were rare.
Nwosu et al. (2021) [52]	Qualitative study; VRG (*n* = 15)	Samsung Gear VR system with options for (1) a 5 min beach relaxation video, (2) a 10 min forest meditation, or (3) a 5 min rollercoaster ride.	High acceptance rate: 93.3% of participants had positive experiences, preferring the forest and beach scenes. Minor issues included headset weight and focus adjustments.
Schrempf et al. (2022) [53]	Pilot study; VRG (*n* = 54)	Oculus Go with TRIPP (TRIPP Inc., Los Angeles, USA, www.tripp.com; accessed on 10 December 2024) software. Two daily sessions: morning (7–8 min) with breathing exercises and evening (10 min) calming sessions with interactive breath visualization.	Statistically significant reductions in anxiety and physical markers: higher contentment (+19.3%), calmness (+16.3%), relaxation (+28.2%); lower heart rate (−1.3 bpm) and respiratory rate (−0.6 bpm; 26.4% reduction in anxiety post-intervention.
O’Gara et al. (2022) [54]	Two-phase study; VRG1 (*n* = 20), VRG2 (*n* = 16), VRG3 (*n* = 13)	Three progressive VR sessions using compassionate mind training (CMT). VR1 familiarized participants with VR; VR2 included breathing exercises; VR3 introduced CMT with a choice of beach, mountain, or forest environments.	Significant mood (POMS) and mental well-being (WEMWBS) improvements across sessions. VR3 showed a consistent beneficial effect and a statistically significant increase in WEMWBS scores from baseline to VR3. The final session significantly reduced stress levels (DASS21, *p* = 0.03).
Burridge et al. (2022) [55]	Qualitative study; VR Group (*n* = 28)	DR.VR Immersive Therapeutic System (DR VR., Rescape Health, https://www.rescape.health/news/drvrfrontline-0; accessed on 10 December 2024) has options for short 7.5 min experiences (e.g., walk on the beach, swim underwater, meditation).	Mean anxiety scores decreased by 40% (*p* < 0.001; mean anxiety score decreased from 4.43 to 2.65), and mean pain scores by 29% immediately post-intervention. All participants rated the experience positively and would recommend it to others.
Kupczik et al. (2022) [56]	Qualitative study; VRG (*n* = 20)	HTC Vive HMD with two immersive experiences: (A) a 360° video of a walk through a sanctuary or (B) interactive woodland and beach scenes with object interaction (e.g., rocks, shells).	A total of 85% preferred the VR experience due to its interactivity and greater realism than the 360° video. Higher immersion scores were linked to the freedom to interact with the environment in VR, which significantly increased participant immersion and engagement.
Moscato et al. (2021) [57]	Feasibility study; home-based palliative care cancer patients (*n* = 25)	VR HMD with individualized content; 30 min sessions tailored to personal interests and comforting environments.	Anxiety and pain levels were measured pre- and post-session, showing a statistically significant reduction in anxiety and improvement in overall well-being.
Kelleher et al. (2022) [58]	Pilot trial; advanced colorectal cancer patients in palliative care (*n* = 15)	VR HMD focused on guided meditative environments for pain and anxiety reduction; 20 min sessions daily.	Pain and anxiety measured pre- and post-session indicating substantial reduction; results support VR as a complementary therapy for pain management.
Greinacher et al. (2024) [59]	Qualitative study; terminally ill cancer patients (*n* = 18)	Interviews exploring patient perspectives on VR; sessions used calming, customized VR content in 30 min blocks.	Interviews revealed positive patient feedback, with VR seen as effective for distraction, emotional relief, and reducing anxiety.
McAnirlin et al. (2024) [60]	Protocol study; hospice patient–caregiver dyads (*n* = 30)	Tandem VR™ (Virtual Nature Lab, https://www.virtualnaturelab.org/tandemvr; accessed on 10 December 2024) using synchronized nature-based content for both patients and caregivers; 30 min sessions.	Psychological measures showed improvements in anxiety and quality of life; also noted reduction in fear of death and enhanced emotional support for caregivers.
Deming et al. (2024) [61]	Prospective study; hospice patients (*n* = 28)	VR with serene nature video content for calming effects; 15 min sessions twice daily.	Anxiety and depression measured with psychometric tools showed a 30% reduction in anxiety scores and improved well-being after consistent VR exposure.

*n* stands for the number of included participants in the study; VRG: VR group.

**Table 5:** Risk of bias assessment.

Study	Randomization Process	Deviations from Interventions	Missing Data	Outcome Measurement	Selective Reporting	Overall Bias	Summary of Justifications
Gerlach et al. (2024) [31]	Low Risk	Low Risk	Low Risk	Low Risk	Low Risk	Low Risk	Clear randomization and adherence protocol. Outcome measures are complete and consistently applied, with no selective reporting identified.
Corvin et al. (2024) [33]	Some Concerns	Low Risk	Low Risk	Low Risk	Some Concerns	Some Concerns	Randomization lacks full transparency. Adherence checks are robust, but selective reporting concerns exist for some secondary measures, potentially impacting outcome interpretation.
Brungardt et al. (2024) [34]	Low Risk	Low Risk	Low Risk	Low Risk	Low Risk	Low Risk	Randomization process clearly documented with strong adherence monitoring. Anxiety and quality of life outcomes fully reported without bias.
Nwosu et al. (2021) [52]	Some Concerns	Some Concerns	Low Risk	Some Concerns	Some Concerns	Some Concerns	The randomization process is not fully detailed, with minor deviations noted. All outcomes are generally reported, though reporting completeness is unclear for some qualitative feedback.
Schrempf et al. (2022) [53]	Low risk	Low risk	Low risk	Low Risk	Some Concerns	Low risk	The study is well designed with robust randomization, high adherence, and consistent outcome measures. Minor concerns about selective reporting were noted, but they do not detract from the study’s overall validity.
O’Gara et al. (2022) [54]	Some Concerns	Low Risk	Low Risk	Low Risk	Low Risk	Some Concerns	Randomization is limitedly transparent. The intervention is consistent across participants, with minimal missing data. However, there are some concerns over selective reporting clarity.
Burridge et al. (2023) [55]	Low Risk	Low Risk	Low Risk	Low Risk	Low Risk	Low Risk	Well-implemented randomization, with robust checks on adherence and precise outcomes. They report full anxiety and pain results pre- and post-intervention without selective reporting.
Kupczik et al. (2022) [56]	Some Concerns	Some Concerns	Low Risk	Low Risk	Low Risk	Some Concerns	Randomization lacks detail, and minor adherence issues were noted. Outcomes are measured thoroughly, though minor selective reporting concerns remain in the feedback results.
Moscato et al. (2021) [57]	Some Concerns	Low Risk	Low Risk	Low Risk	Some Concerns	Some Concerns	Limited transparency in randomization process, but adherence is well managed. Outcome measures are consistent; some concerns over selective reporting due to limited secondary outcome detail.
Kelleher et al. (2022) [58]	Low Risk	Low Risk	Low Risk	Low Risk	Some Concerns	Low Risk	Strong randomization and outcome reporting. Minor concerns regarding selective reporting of secondary outcomes, but primary results are clear and well-supported.
McAnirlin et al. (2024) [60]	Some Concerns	Low Risk	Low Risk	Low Risk	Some Concerns	Some Concerns	Limited details on randomization; consistent adherence and outcome measurement, though selective reporting on some secondary outcomes. Results are reliable for primary measures.
Deming et al. (2024) [61]	Low Risk	Low Risk	Low Risk	Low Risk	Low Risk	Low Risk	Thorough randomization and adherence tracking. Full reporting on anxiety and depression measures with no bias or selective reporting noted.
Guenther et al. (2022) [62]	Some Concerns	Low Risk	Low Risk	Low Risk	Some Concerns	Some Concerns	Some concerns in randomization detail and selective reporting were noted. Adherence is high, and outcome measures are reliably assessed with consistent reporting of results.

Low Risk: Clear, adequately reported with minimal bias risk. Some Concerns: Minor potential for bias due to lack of detail or minor inconsistencies.

## Data Availability

The original contributions presented in this study are included in the article. Further inquiries can be directed to the corresponding author.
